# Method Validation and Characterization of Red Pigment in *Beta vulgaris* Peels and Pomaces by HPLC-UV and UHPLC-MS/MS

**DOI:** 10.1155/2022/2229500

**Published:** 2022-08-23

**Authors:** Vincent Rotich, Phanice Wangila, Jackson Cherutoi

**Affiliations:** ^1^Department of Chemistry & Biochemistry, School of Sciences and Aerospace Studies, Moi University, P.O. Box 3900 30100, Eldoret, Kenya; ^2^Africa Centre of Excellence in Phytochemicals, Textile and Renewable Energy (ACEII PTRE), Moi University, P. O. Box 3900 30100, Eldoret, Kenya; ^3^Department of Physical Sciences, School of Science & Technology, University of Kabianga, P.O. Box 2030 20200, Kericho, Kenya

## Abstract

Color pigments from plant, animal, or mineral sources can be identified, separated, and quantified for various purposes. It is expected that pigments from *Beta vulgaris* L. peels and pomaces could be used to develop natural dyes that can find applications in areas such as food or textile dyeing industries. This work aimed at identifying and quantifying the pigment in the *B. vulgaris* L. peels and pomaces extracts as well as validating the method by high-performance liquid chromatography combined with ultraviolet spectroscopy (HPLC-UV) and ultra-high-performance liquid chromatography coupled with triple quadrupole (TSQ) mass spectrometry (UHPLC-MS/MS). Column chromatography was used to isolate compounds after methanolic solvent extraction. Identification and quantification of the pigments in the extract were achieved using reverse-phase HPLC with a UV detector (538 nm). The UHPLC-MS/MS was used for further confirmation of colored compounds in the extract. Method validation included the use of betanin standard (betanidin 5-*β*-D-glucopyranoside), determination of repeatability (precision), calibration curve linearity, and sensitivity (LOD and LOQ) tests. Betanin was detected in the sample at retention times of 7.699 and 7.71 minutes, respectively, which closely matched the *t*R (7.60 min) of the standard, according to HPLC-UV and LC-MS/MS data. The average betanin concentration was 3.81 0.31 mg/g of dry weight, according to the HPLC-UV analysis. The LC-MS/MS data revealed the existence of several compounds, including betanin (4.31 ± 2.15 mg/g), isobetanin (1.85 ± 2.20 mg/g), 2, 17-bidecarboxy-neobetanin (0.71 ± 0.02 mg/g), betanidin (0.71 ± 0.03 mg/g), 2-*O*-glucosyl-betanin (0.40 ± 0.10 mg/g), and isobetanidin (0.36 ± 1.26 mg/g), among other compounds whose yields were too low. In conclusion, the peels and pomaces of *B. vulgaris* L. can be a useful source for the extraction of a red dye for use in coloring, such as the dyeing of textile substrates.

## 1. Introduction

Peels and pomaces of *B. vulgaris* L. have a dark reddish-purple physical appearance that is a result of the presence of phytochemical pigments, making them promising, sustainable biowaste dye sources. It contains highly active coloring pigments such as betalains [1, 2] including red-violet color betacyanins and yellow-orange color betaxanthins [3]; carotenoids; water-soluble vitamins [4] as well as high concentrations of nitrate (644 to 1800 mg/kg) [5]. Betanin (betalains) pigments are the main components of *B. vulgaris* L. and are known to impart their red color [6, 7] rendering beetroot the richest source of betalains [8]. According to the literature, *B. vulgaris* L. also contains modest amounts of several beneficial substances as betaine, folate, and glycine [4].

Natural dyes have been the primary color additives for numerous uses over the millennia up to the 19th century [9, 10]. However, the development of synthetic dyes manufactured by humans has nearly entirely replaced natural dyes in the textile business. While their synthetic counterparts offer the whole spectrum of color strength, wash, and light fastness at comparatively low costs, the majority of natural dyes have poor to moderate wash and light fastness [11]. Additionally, they display advantageous application qualities with a broad selection of colors to choose from, increased repeatability, and improved dyeing quality [12]. However, the majority of synthetic dyes are created using hazardous intermediate compounds and basic ingredients (heavy metals and azo groups) [13–15] and are consequently risky, cancer-causing, and environmental hazards. They contribute to severe health problems, disruption of the ecological balance, global warming, ozone layer depletion, and water contamination [16, 17]. As a result, both researchers and businesses are interested in the development of natural dyes that are nonallergic, nontoxic, and beneficial to the environment [15, 18–21].

The global demand for natural dyes to mitigate the adverse effects of synthetic dyes has necessitated the advancement of studies on them. Natural dyes are among the promising options for developing greener dyeing processes. This is demonstrated by the rise in recent research in fields including eco-driven design, green clothes, and sustainable clothing, such as the coloring of bacterial cellulose fiber [22]. Being derived from a natural source, the dye from *B. vulgaris* L. peels and pomaces is thought to be noncarcinogenic and have no adverse impact on the environment or human health [19]. Additionally, the easily accessible and low-cost plant wastes (peels and pomace) can be used in the production of dyes for textile dyeing. The exploitation of *B. vulgaris* L. waste in this study will increase knowledge in natural dye isolation, separation, identification, and quantification in plant species.

There have been several attempts to create techniques for the quick separation and characterization of bioactive compounds, and several HPLC-based approaches have been published for the investigation of these compounds in crude extracts [23]. Given the significance of bioactive substances, such as pigments in plants, it is imperative to develop a quick and accurate method for separating, identifying, and quantifying these substances. Due to their great accuracy and adaptability, HPLC and UHPLC are the best methods to use for the analysis of chemicals in plants. These techniques have essential developments for efficient separations and identification, such as hyphenated systems [24]. The HPLC method combined with a UV photodiode array detector (DAD), for instance, offers greater structural details on the substances. Due to its dependability in regular analyses of plant chemicals, the reversed-phase (RP) HPLC system on a C18 column with a binary mobile phase containing acidified water and a polar organic solvent such acetonitrile or methanol is the most popular HPLC system [24]. Additionally, due to its effective resolution, LC-MS/MS is among the most trustworthy methods for the identification and characterization of chemicals [24]. Such chromatographic techniques do, however, have certain inherent drawbacks, such as coelution and prohibitive cost.

This study aimed at the characterization of dye from *B. vulgaris* L. peels and pomaces by HPLC-UV and LC-MS/MS to further elucidate the structural compounds [25]. In order to separate, identify, and quantify the red pigments in the extract, a validated HPLC-UV and LC-MS/MS method has been developed. This approach applies the quality control techniques of repeatability (precision), linearity, and sensitivity. The linearity of the calibration curve and Passing–Bablok regression plot served as evidence that the two procedures for extract analysis of *B. vulgaris* L. peels and pomaces were in agreement with one another.

## 2. Materials and Methods

### 2.1. Chemicals and Reagents

Acetonitrile, acetone, ethyl acetate, hexane, isopropyl alcohol, methanol, formic acid, and ammonium acetate were acquired from Loba Chemie, India. Betanidin 5-*β*-D-glucopyranoside standard with percent purity ≥ 95% was used (Wuhan ChemFaces Biochemical Co., Ltd, China). Sodium formate and 2, 2-difluoroethoxy phosphazene were used (Fisher Scientific, USA). Distilled and deionized water, as well as cleaned-up samples (before HPLC analysis), were filtered through a 25 mm and 0.45 mm nylon syringe filter (Millipore, USA). The analytical grade of each chemical or reagent purchased allowed for usage without further purification.

### 2.2. Equipment

Equipment used is as follows: cellulose extraction thimbles from Whatman and a Soxhlet apparatus (Sigma Aldrich, Germany). Traditional Portable PX Meter (0.1 pH Resolution). TLC silica gel 60 (Merck, Germany). LED UV lamp (New Lights, China). Rotational vacuum evaporator (Hahnvapor Rotary Evaporator HS-2005S, Korea). SPD-20 A UV detector and column (Supelcosil LC-18, 5 UM, Dimension: 15 cm by 4 mm, USA) are used with HPLC (LC-10AT VP Shimadzu). A Vanquish Horizon UHPLC with a Thermo Scientific Hypersil GOLD aQ™ column (50 × 2.1 mm, 1.9 *μ*m) is connected to a Thermo Scientific Triple Quadrupole Mass Spectrometer (TSQ) Altis that is equipped with an OptaMax NG.

### 2.3. Collection and Preparation of Samples

Fresh *B. vulgaris* L. plant peels and pomace were procured from food establishments and fruit juice vendors in Eldoret town, Kenya, and afterwards stored at −4°C. After being cut into small pieces, the samples were dried in an oven at 40°C for 8 to 10 hours until they were totally dry and no more change in weight. A pestle and mortar were used to pound the material into a fine powder. Using sieve No. 100, sieving was done to get a very fine powder (around 0.15 mm). The Soxhlet extraction method was then used to extract 20 grams of the sample using reflux in 200 mL of methanol for 11 hours, following Rotich, Wangila, and Cherutoi's optimized procedure [26]. According to Antigo, Rita de Cássia, and Grasiele [27], a few drops of formic acid were added to maintain the pH between 4 and 5 in order to retain the thermal and pH stability of the red color of *B. vulgaris* L. peels and pomaces. The finest solvent for the extraction of natural dyes, according to reports, is methanol, which is renowned for producing high absorbance values extracts [28, 29]. Once it had been extracted, the dye was concentrated using a vacuum rotary evaporator to produce 10.65 g (53.25% of dry weight) of solid crude extract. This was done by filtering the dye through Whatman No. 1 filter paper.

### 2.4. Thin Layer and Column Chromatography

Different combinations and ratios of multiple solvents (from polar to nonpolar) were mixed, volume by volume, and utilized on TLC plates in order to establish the optimal solvent system for isolation of chemicals in the extract by column chromatography [26]. Deionized water, methanol, acetonitrile, isopropyl alcohol, and glacial acetic acid (a protic solvent) were utilized as polar solvents. Hexane served as a nonpolar solvent, and acetone and ethyl acetate served as intermediates. The plate was removed once the solvent reaches approximately 1 cm from the end and then put into a UV LED chamber (365 nm and 254 nm) for observation. In order to determine the R*f* (retardation factor) values (Table 1) using equation (1), the acquired color was then circled with a pencil, and their distance traveled was recorded.

(1) $$\begin{array}{c} Rf=\frac{\text{Distance spot travels}\;\left(\text{cm}\right)\times 100}{\text{Distance solvent travels}}. \end{array}$$

The best solvent system established constituted methanol: acetic acid (glacial): distilled water at (90 : 7 : 3) % ratio, respectively, as observed in Table 1. To isolate the extract, this mixture was employed in column chromatography packed with silica gel (60–200 mesh) and eluted at 0.6 mL/min [30]. After being concentrated, the obtained fractions on the amber vials were examined further.

### 2.5. Conditions for LC-MS/MS and HPLC-UV

To create the standard stock solutions for the calibration curves, the betanin standard (0.1 g) was first dissolved in 100 mL of MeOH solution. On the other hand, MeOH (100 mL) was used to dissolve the pure sample extract powder (0.1 g). A 2-percent HCl and MeOH solution was used to make up the volume (10 mL) after a 1 mL aliquot was weighed in a 10 mL volumetric flask. After filtering the sample with a 0.45 *μ*m nylon syringe into HPLC amber vials, the HPLC equipped with a UV detector at 538 nm and Supelcosil LC-18 column was used to analyze the sample in triplicates. Pressure at 107 bar, isocratic acetonitrile (70%), and ammonium acetate buffer (0.7 mM) in water at 30% as the mobile phase, at a flow rate of 0.4 mL/min, and an injection volume of 10 *μ*L were the settings specified. The column was kept at a constant temperature of 35°C. In this instance, chromatograms were produced using data analysis software (Shimadzu Lab Solutions Version 5.60SP2).

Thermo Scientific TSQ Altis equipped with an OptaMax NG and connected to a Vanquish Horizon UHPLC was used for the LC-MS/MS analysis. The UHPLC method was used as follows: with 0.1 percent formic acid in H_2_O as solvent A and 0.06 percent formic acid in MeOH as solvent B, an isocratic gradient of 10% B for 10 min, 10%–100% B for 30 min, and 100% B for an additional 10 min, using a flow rate of 0.3 mL/min; 5 *μ*L injection volume; and MS detector. Thermo Scientific's Hypersil GOLD aQ™ column (50 × 2.1 mm, 1.9 *μ*m) was used for the separation, and the mass to charge ratio (*m/z*) 50–1800 scan mode was used for MS acquisition. The MS/MS settings included a 4500 V capillary voltage, 1.6 bar nitrogen gas nebulizer, 200°C ion source temperature, 7 L/min dry gas flow, and 3 Hz and 10 Hz spectral rates for the first mass spectrometer (MS1) and the second mass spectrometer (MS2), respectively. The 10 strongest ions per MS1 were chosen for subsequent CID with stepped CID energy applied in order to acquire MS/MS fragmentation. As previously described by Garg et al. [30] and Wolf et al. [31], the employed tandem MS parameters were put into practice. The lock mass was 2, 2-difluoroethoxy phosphazene, and sodium formate was employed as an internal calibrant. The TraceFinder version 4.1 software was used to process all the data.

### 2.6. Method Validation

#### 2.6.1. Repeatability (Precision)

The accuracy of the results was determined by the close retention times of the betanidin 5-*β*-D-glucopyranoside standard solution at the five concentrations of 10 ppm, 15 ppm, 20 ppm, 27 ppm, and 35 ppm in five replicates at different times on day 1 and the same replicate concentrations at different times on day 2 for intraday repeatability (same day) and different days (interday) precision. The corresponding retention times and peak regions were tabulated.

#### 2.6.2. Linearity in Calibration Curve

A 1 mL aliquot of the stock solution was diluted to concentrations of 10, 15, 20, 27, and 35 ppm before being injected into the HPLC-UV system to create a five-point calibration curve for the standard. The correlation coefficient *R*^2^ was 0.9928. It had a good prediction rate of >0.96.

The analytical curve for LC-MS/MS was built using concentrations between 10 and 50 ng/mL. Weighted (1/concentration) least-squares regression analysis was used to create calibration curves, which were then fitted to the Passing and Bablok curves. Standards and samples for calibration curves were prepared for analysis in five replicates. The Passing and Bablok curve's *R*^2^ value was 0.9988, which was also >0.96 and predicted trustworthy outcomes.

#### 2.6.3. Sensitivity

Limit of detection (*LoD*) and limit of quantification (*LoQ*) were used to gauge the method's sensitivity. By evaluating the signal-to-noise ratio from a low concentration of the standard analyte and contrasting it with the baseline peak of a blank sample, the *LoD* and *LoQ* were calculated. On the HPLC-UV and LC-MS/MS, triplicate 1, 0.8, 0.6, 0.4, and 0.2 ppm samples of the betanin standard were injected. The *LoQ* was established as the minimal concentration at which the peaks could be successfully quantified, which is typically three times the noise level, and the *LoD* as the lowest concentration at which a peak was observed correlating to the projected retention time.

In the LC-MS/MS analysis, the *LoQ* was restricted to ± 20%. The baseline noise approach was used to estimate the *LoQ*. It was projected to have a five signal-to-noise ratio of five. Through the experimental administration of six injections of the standard at the *LoQ* concentration, the *LoQ* was ascertained. Equations (2) and (3) were used to determine the *LoD* and the *LoQ*:

(2) $$\begin{array}{c} LoD=\frac{3S_{a}}{b}, \end{array}$$

(3) $$\begin{array}{c} LoQ=\frac{10S_{a}}{b}, \end{array}$$

where *S*_*a*_ is the regression line's standard deviation, and *b* is the derived calibration curves' slope.

### 2.7. Betanin Identification by HPLC and LC-MS/MS

This study's major goal was to determine the principal peak seen in the peels and pomace extract of *B. vulgaris* L. by comparing it to the commercially available betanin standard using HPLC and LC-MS/MS. By applying their peak areas and average concentrations while accounting for their standard deviation, the chromatograms of the sample in triplicate were utilized to determine the concentration of the unknown sample. As there were no existing standards for the other chemicals, they were discovered and compared using the literature.

### 2.8. Betanin Quantification

Using the external standard (ESTD) method of quantification and equation (4), the betanin concentration was determined from HPLC-UV data and expressed in mg/g.

The betanin content was quantified from HPLC-UV data using the external standard (ESTD) method of quantification by applying equation (4) and expressed in mg/g.

(4) $$\begin{array}{c} \frac{\left(A\times \text{DF}\right)\times \text{CF}}{S_{\text{wt}}}, \end{array}$$

where *A* is the amount of betanin (mg/mL) expressed as the external standard equivalent (betanidin 5-*β*-D-glucopyranoside standard) from the calibration curve, DF is the dilution factor for the extract, CF is the molecular weight correction factor to convert the betanin (mg/g) calculated as the external standard equivalents to their respective forms, and *S*_wt_ is the initial sample weight (g)·(g) [3].

## 3. Results and Discussion

### 3.1. Standard Calibration Curve Linearity and Range

**Figure 1:**
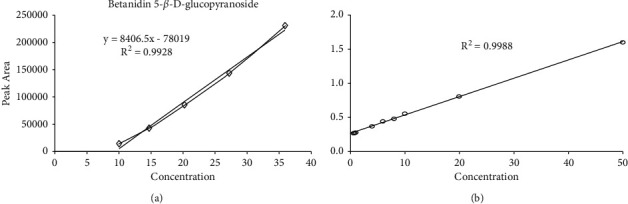
(a) Preparative HPLC calibration curve for betanidin 5-*β*-D-glucopyranoside standard; (b) passing and Bablok fit of betanidin 5-*β*-D-glucopyranoside standard in *B. vulgaris* L. peels and pomaces extracts determined with TSQ-MS.

The calibration graph regressed as the *x* and *y* axes (Figure 1(a)) had a linear relationship, which led to a high positive correlation between the concentration of the betanin standard (*x*) and the signal (*y*). A further illustration of the reproducibility of betanin qualifying in *B. vulgaris* L. peels and pomace extracts evaluated with TSQ LC-MS is shown in Figure 1(b), which also demonstrates the method's robustness in routine qualitative and quantitative determinations.

### 3.2. Repeatability (Precision)

Repeatability or precision in chromatography is a way of depicting the closeness of the results obtained with the same sample or standard following the same process: the procedure, operators, measurements, operating conditions, and location over a certain period, that is, intraday (within one day) and interday (between days). The results showed that the mean *t*R (7.670 ± 0.15 min), peak areas ((28878 ± 0.11), and quantity estimates (12.71 ± 0.10 mg/g) of intraday were close to the mean *t*R (7.655 ± 0.24 min), peak areas (28129 ± 0.22), and quantity estimates (12.63 ± 0.27 mg/g) of interday of the betanidin 5-*β*-D-glucopyranoside standard (Table 2). The small changes in *tR* were within the acceptable range of <0.5 min. The precision of the quantification of betanin in *B. vulgaris* L. peels and pomaces extracts was fairly satisfactory, with the difference between the highest and smallest values in the different runs being less than 10% for all samples.

### 3.3. Instrumental Sensitivity

The instrument software (Shimadzu Lab Solutions Version 5.60SP2) determined that the analyte betanidin 5-*β*-D-glucopyranoside standard at 0.2 ppm (as per equation (2)) had a signal-to-noise ratio of 3 : 1, making this the lowest concentration at which the analyte could be identified in the HPLC-UV. Furthermore, LC-MS/MS was successful in achieving 1.21 ng/mL *LoD*. Equation (3) was used to get the *LoQ*, which was found to be 0.8 ppm and 3.68 ng/mL for HPLC and LC-MS/MS, respectively. The instrumental software determined the signal-to-noise ratio in the LC-MS case to be 10 : 1, which was within the permitted range for HPLC and LC-MS/MS analysis.

### 3.4. Identification of Betanin

**Figure 2:**
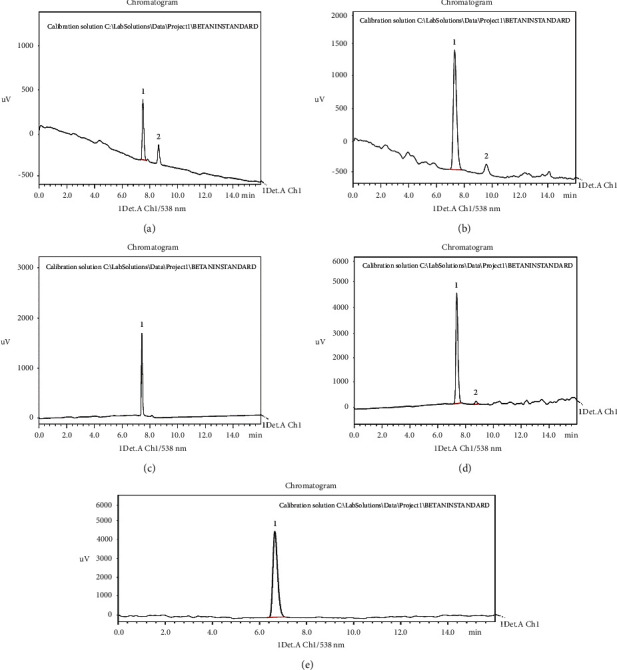
Chromatograms for the betanin standard (betanidin 5-*β*-D-glucopyranoside) at 10 ppm (a), 15 ppm (b), 20 ppm (c), 27 ppm (d), and 35 ppm (e).

The HPLC chromatogram in Figures 2(a)–2(e) of betanin standard at various concentrations (10, 15, 20, 27, and 35 ppm respectively) exhibits an exceptional peak (peak 1), attributed to betanin in the standard (betanidin 5-*β*-D-glucopyranoside standard), which is in charge of giving *B. vulgaris* L. peels and pomaces their red color [32]. In Figures 2(a) and 2(b), a second peak (ghost peak) can be seen after the total elution time of initial betanin standard injections. The ghost peak most likely came from the HPLC system injector that was vibrating, but it was resolved; hence, relatively smooth peaks were observed in the subsequent injections.

**Figure 3:**
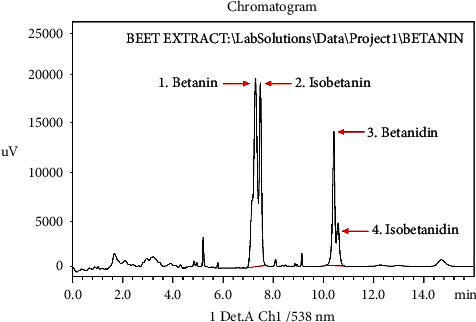
HPLC-UV chromatogram for the *B. vulgaris* L peels and pomaces sample with UV detector at 538 nm.

The chromatographic pattern of the *B. vulgaris* L. peels and pomaces sample showed four distinct peaks at *t*Rs of 7.699 ± 0.10, 7.890 ± 0.00, 10.065 ± 0.31, and 10.008 ± 0.27 min, respectively (Figure 3). By comparing its *tR* with the generated chromatograms of the betanin standard, peak 1 in the plant sample could be located. The betanin peak, which was measured at a *tR* of 7.699 ± 0.10 min, was consistent with the betanin standard's results, which showed an essentially identical peak at 7.662 min.

Peak 2 was most likely equivalent to isobetanin in the *B. vulgaris* L. peels and pomaces sample, while peaks 3 and 4 were most likely betanidin and isobetanidin, respectively, based on their longer retention durations that were allocated based on their *t*Rs from prior studies [32].

*B. vulgaris* L. peels and pomaces exhibited a much greater betanin relative peak area compared to other compounds, hence distinguishing it from other compounds in the sample [3]. The betanin, isobetanin, betanidin, and isobetanidin molecules represented 38.19 ± 0.88, 29.60 ± 0.24, 20.10 ± 0.51, and 12.11 ± 1.3%, respectively, of the total betalain peak area (Table 3). Furthermore, their respective peak areas were directly proportional to their height (relative abundance), and it is observed that betanin was more abundant (21953.00 ± 0.91), followed by isobetanin (21659.67 ± 0.72), betanidin (15596.33 ± 0.23), and isobetanidin (5593.00 ± 0.67), respectively, in that sequence [3].

### 3.5. Estimation of the Betanin Content in the *B. vulgaris* L. Peels and Pomaces

The amount of betanin present in the plant sample was expressed in mg/g using the calibration curve's external standard equivalent (betanidin 5-*β*-D-glucopyranoside standard). An average of 3.81 ± 0.30 mg/g of betanin concentration was found by the HPLC-UV analysis of the sample fractions (Table 4). The result of converting to mg/100 g of dry weight was 381.00 30 mg/100 g betanin. The results corroborated those of Prieto-Santiago, Cavia, Alonso-Torre, and Carrillo [33] who determined that the total betanin concentration of red beet ranged from 250 to 850 mg/100 g of dry weight.

Table 5 lists the betalain compounds' estimated percentage contributions to the total betalain content (TBC) of the peels and pomaces of *B. vulgaris* L. Betanin and isobetanin made up the majority of the betalains, accounting for 38.16 ± 0.31% and 29.62 ± 0.22%, respectively, of the plant extract's total betalain composition. The findings supported those of Kathiravan et al. [6] in their investigation into the antioxidant, pigment, and microbial inactivation of Ready to Drink (RTD) beetroot (*B. vulgaris* L.) juice. The results demonstrated that betanin is the primary compound present in high quantities in the red *B. vulgaris* L. peels and pomaces giving it red coloring. The results of this study enhance the significance of processing *B. vulgaris* L. peels and pomaces for the production of pigments, due to its red-violet betalain compounds, as supported by Khan et al. [34] who investigated *Rivina humilis* L. berries as a potential source of betalains for pigment identification, nutritional composition, bioactivity, and in vitro cancer cell cytotoxicity.

### 3.6. LC-MS/MS Analysis

**Figure 4:**
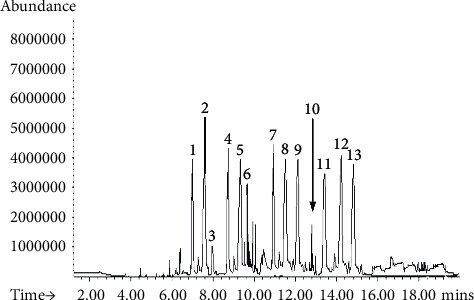
The UHPLC chromatogram for *Beta vulgaris* L peels and pomace sample.

The LC chromatographic retention times of various compounds in the *B. vulgaris* L. peel and pomaces sample are represented in Figure 4. It was observed that different peaks were obtained at different *tR*. The highest peak (high relative abundance) is at the *tR* of 7.71 mins. This is followed by peaks at *tR* sequence of 10.93, 8.81, 14.78, 9.46, 11.67, 12.13, 6.97, 14.86, 14.18, 9.68, 13.72, and 7.98 minutes. The peaks are attributed to the compounds 2-*O*-glucosyl-betanin, betanin, 2-*O*-glucosyl-isobetanin, isobetanin (isobetanidin 5-glucoside), betanidin, ferulic acid hexoside, 17-decarboxy-neobetanin, isobetanidin (17–decarboxy-betanidin), isobetanidin (17–decarboxy-betanidin), neobetanin, 17-decarboxy-betanin, 2, 17-bidecarboxy-betanin (isobetanin), and 2,17-bidecarboxy–neobetanin and isovitexin (apigenin 6-C- glucoside), respectively. The betanin standard's (betanidin 5-*β*-D-glucopyranoside) *t*R was found to be 8.189 minutes.

**Figure 5:**
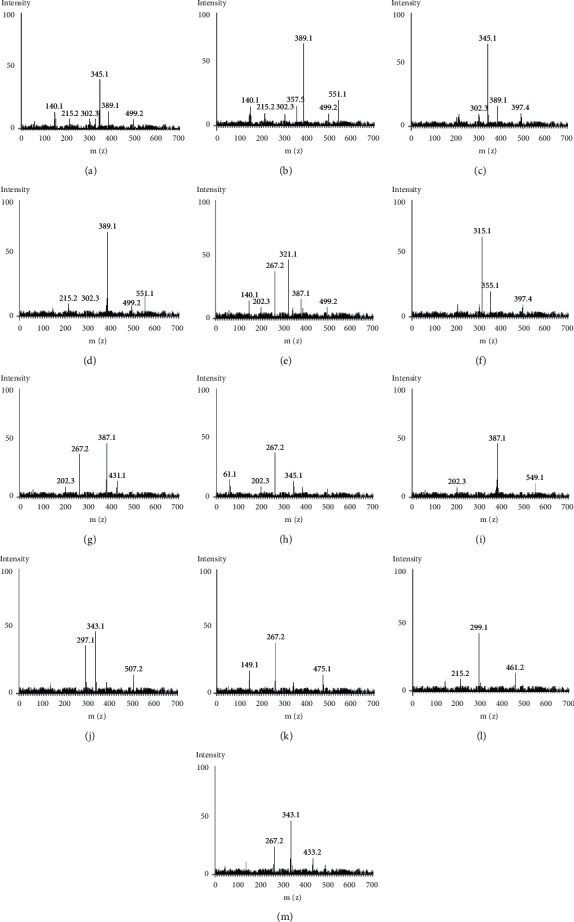
MS-MS spectra of the betalains compounds identified in *Beta vulgaris* L peels and pomaces extract; (a) 2-O-glucosyl-betanin; (b) betanin; (c) 2-O-glucosyl-isobetanin; (d) isobetanin (isobetanidin 5-glucoside); (e) betanidin; (f) ferulic acid hexoside; (g) 17-decarboxy-neobetanin; (h) isobetanidin (17 –decarboxy-betanidin); (i) neobetanin; (j) 17-decarboxy-betanin; (k) 2, 17-bidecarboxy-betanin (isobetanin); (l) 2,17-bidecarboxy–neobetanin, and (m) isovitexin (apigenin 6-C- glucoside).

The MS-MS spectrum of each betalain compound in the *B. vulgaris* L. peels and pomaces sample is shown in Figure 5. The protonated 2-*O*-glucosyl-betanin, 2-*O*-glucosyl-isobetanin, and isobetanin (isobetanidin 5-glucoside) molecules (peaks 1, 3, and 4) resulted probably in loss of glucose to achieve *m*/*z* of 345.1, 389.1, and 345.1, respectively (Table 6). The betanin molecule (peak 2), with *m/z* of 551.11 [*M* + *H*]^+^, emanated to probably loss of glucose and eventual formation of the other twelve compounds that are part of its degradation [3, 35] and further fragmentation to *m*/*z* 389.1 [*M* + *H*]^+^. The *m/z* of betanin and isobetanin is comparable; however, their *t*Rs are different, measuring 7.71 and 8.81 minutes, respectively. As demonstrated by Slatnar, Stampar, Vebaric, and Jakopic [3] in HPLC-MS^n^ identification of betalains profile in different parts and cultivars of beetroot, peaks 1 and 3 might have been having similar epimers, thus similar ion scan of *m/z* 389.1 [*M* + *H*]^+^ but different *t*Rs, and possibly corresponded to 2-*O*-glucosyl-betanin and 2-*O*-glucosyl-iso (*Beta vulgaris* L. ssp. *vulgaris*).

Given the prior information [32], the *m/z* of 387.1 [*M* + *H*]^+^ may correspond to betanidin (peak 5), and subsequent fragments with *m/z* 321.1 and 267.2 [*M* + *H*]^+^ were formed. Ferulic acid hexoside, discovered as a compound at peak 6 with a *m/z* of 355.1[*M* + *H*]^−^, is also indicated to be present in trace amounts in *B. vulgaris* L. peels and pomaces extract. Ferulic acid lost carbon dioxide as a result of the deprotonated molecule, which produced the radical ion [*M* − *H*]^−^, as the distinctive fragment ion with a *m/z* of 315.1[*M* − *H*]^−^. This demonstrates that free glucosides of ferulic acid, which are glucosides, are present in the *B. vulgaris* L. peels and pomaces extract. Ferulic acid hexoside function as intermediaries in the biosynthesis of betanin [3, 35].

Peak 7 corresponded to 17-decarboxy-neobetanin pseudomolecular ions observed at *m*/*z* of 431.1 [*M* + *H*]^+^ that furnished the protonated fragment ions having *m*/*z* of 387.1 and 267.2 [*M* + *H*]^+^. Peak 8's ion scan, which has a mass of 345.1 [*M* + *H*]^+^, is thought to be isobetanidin (17-decarboxy-betanidin), and it produced a protonated ion with a mass of 267.1 [*M* + *H*]^+^. Neobetanin, which was discovered in peak 9 with a *m/z* of 549.1 [*M* + *H*]^+^, is one of the byproducts created during the production process' isolation of betanin. It further fragmented to yield ion with *m*/*z* of 387.1 [*M* + *H*]^+^ depicting further glucose loss [3].

The other betanin breakdown products discovered are 17-decarboxy-betanin, 2, 17-bidecarboxy-betanin, and 2, 17-bidecarboxy-neobetanin. These compounds have *m/z* values of 507.2 [*M* + *H*]^+^, 475.1 [*M* − *H*]^−^, and 461.2 [*M* + *H*]^+^, respectively, and are connected as peaks 10, 11, and 12, respectively [32]. While the peak 10 molecule fragmented to produce protonated ions at *m/z* of 343.1 *M* + *H*]^+^ and 297.1 [*M* + *H*]^+^, the molecule at peak 11 produced a deprotonated molecule [M − H]^−^ at *m/z* 267.1 that was likely caused by the loss of carbon dioxide. Peak 12's shattered molecules produced a protonated 299.1 [*M* + *H*]^+^ ion, indicating possible glucose loss. Peak 13's molecular ion at 433.2 [*M* + *H*]^+^ has a similar mass to isovitexin (apigenin 6-C-glucoside). It produced [*M* + *H*]^+^ fragment ions with *m/z* values of 343.1 and 267.2. This discovery established the flavanol status of isovitexin, confirming that the samples of *B. vulgaris* L. peels and pomaces contained flavanols as bioactive substances [3, 32].

Eleven of the thirteen compounds were thought to be betalain compounds. The highest concentration of betalains is found in *B. vulgaris* L, which contains betalains (also known as betacyanins) in a ratio of almost 93% of its total bioactive chemicals [8]. Betanin and isobetanin were observed to be the predominant betalains in the *B. vulgaris* L. peels and pomace sample evidenced by their high relative abundance (4.31 ± 2.15 mg/g and 1.85 ± 2.20 mg/g of dry weights respectively). Betanin represented 43.12% of the total betalain peak area (TBPA), which is approximately 4.31 ± 2.15 mg/g of dry weight, while isobetanin represented 18.45% or approximately 1.85 ± 2.20 mg/g of *B. vulgaris* L. dry weight (Table 5), corroborating Slatnar et al. [3] findings that betanin represented a greater percentage of TBPA in beetroot peels in his study. The other relatively abundant betalains are 2, 17-bidecarboxy-neobetanin (7.07% of TBPA) and betanidin (7.05% of TBPA) constituting approximately 0.71 ± 0.02 mg/g and 0.71 ± 0.03 mg/g of *B. vulgaris* L. peels and pomace dry weights, respectively. In comparison to HPLC-UV, the LC-MSMS approach has shown to be more accurate and sophisticated due to the observation of more compounds. The findings of this work show that HPLC-UV and LC-MSMS are comparable tools for separating, identifying, and quantifying a variety of phytochemicals from plants or any other natural source [36], such as when processing the peels and pomace of *B. vulgaris* L. to make dyes or pigments.

## 4. Conclusion

The HPLC-UV calibration curve of the different concentrations of betanidin 5-*β*-D-glucopyranoside betanin standard and the LC-MS/MS Passing and Bablok curve had *R*^2^ values > 0.96, confirming their linearity, hence the robustness of the two methods. The high repeatability and sensitivity achieved were indicative of satisfactory accuracy of the quality control and validation process. The retention time for the betanin compound in the sample extract was found to be 7.699 and 7.71 minutes for HPLC-UV and LC-MS/MS, respectively. Betanin content was quantified to be approximately 3.81 ± 0.31 mg/g of the dry weights of *B. vulgaris* L. peels and pomaces in the HPLC-UV method. Comparatively, LC-MS/MS method also demonstrated that betanin is the most abundant compound in the extract about 31 ± 2.15 mg/g of its dry weight. The results of this study have attested that the content and composition of betalains that are responsible for the red-violet color in *B. vulgaris* L. peels and pomaces can be established through HPLC-UV and LC-MS/MS methods. The suggested methods have proven to be applicable in comparative analysis, hence suitable for the identification and quantification of pigments in plant sources. Therefore, they are recommended for application in the production of natural pigments for use in coloration (food or textile dyeing) to mitigate the adverse effects of synthetic dyes.

## Figures and Tables

**Table 1:** Component of mobile phase used in TLC analysis of *B. vulgaris* L. peels and pomaces.

Component of mobile phase	Ratio (v/v)	*Rf*
Hexane	1	0.02
Methanol	1	0.14
Hexane : methanol	8 : 2	0.17
Hexane : methanol	3 : 7	0.21
Acetone : ethyl acetate	6 : 4	0.13
Acetone : ethyl acetate	7 : 3	0.16
Acetone : ethyl acetate	3 : 7	0.20
Acetone : hexane	7 : 3	0.19
Acetic acid (glacial) : methanol : water (distilled)	0.7 : 9 : 0.3	0.54
Acetic acid (glacial) : methanol : water (distilled)	1 : 6 : 3	0.41
Acetic acid (glacial) : ethanol : isopropyl : water (distilled)	1 : 2 : 6 : 1	0.32
Acetic acid (glacial) : ethanol : isopropyl : water (distilled)	1 : 4 : 3 : 2	0.29
Acetic acid (glacial) : acetonitrile : methanol : water (distilled)	0.5 : 2 : 7 : 0.5	0.23

**Table 2:** Intraday and interday repeatability at various concentrations.

Intraday repeatability					Interday repeatability			
#	Standard sample concentration (ppm)	Retention time, *tR*^a^ (min)	Peak area	Quantity estimates (mg/g)	Standard sample concentration (ppm)	Retention time, *tR*^a^ (min)	Peak area	Quantity estimates (mg/g)
1	10	7.685 ± 0.02	12181 ± 0.01	10.73 ± 0.17	10	7.588 ± 0.42	14029 ± 0.67	10.95 ± 0.06
2	15	7.680 ± 0.01	19543 ± 0.03	11.61 ± 0.02	15	7.592 ± 0.53	19682 ± 0.04	11.62 ± 0.01
3	20	7.602 ± 0.13	37832 ± 0.14	13.78 ± 0.06	20	7.612 ± 0.03	19756 ± 0.l1	11.63 ± 0.87
4	27	7.692 ± 0.25	37004 ± 0.23	13.68 ± 0.22	27	7.794 ± 0.07	37098 ± 0.03	13.69 ± 0.34
5	35	7.689 ± 0.33	37831 ± 0.16	13.77 ± 0.01	35	7.691 ± 0.14	50082 ± 0.25	15.24 ± 0.05
Mean, $\bar{x}$		7.670 ± 0.15	28878 ± 0.11	12.71 ± 0.10		7.655 ± 0.24	28129 ± 0.22	12.63 ± 0.27

Results are the mean values from five replicates ± *S*.D. The different letter “a” means that the *t*R of individual concentrations and days are statistically different.

**Table 3:** Chromatographic peak tables for the plant extract.

Peak #	Compound	*tR*	Peak area	Peak area %	Height
1	Betanin	7.699 ± 0.10	242256 ± 0.16	38.19 ± 0.88	21953.00 ± 0.91
2	Isobetanin	7.890 ± 0.00	187806 ± 0.12	29.60 ± 0.24	21659.67 ± 0.72
3	Betanidin	10.065 ± 0.31	127506 ± 0.66	20.10 ± 0.51	15596.33 ± 0.23
4	Isobetanidin	10.008 ± 0.27	76844 ± 0.34	12.11 ± 1.30	5593.00 ± 0.67

The results are shown as the mean values of three replicates ± SD.

**Table 4:** Betanin content estimates for the *B. vulgaris* L. sample.

Run #	Betanin *t*R	Betanin peak area	Betanin content (mg/g)
1	7.602	213249 ± 0.02	3.47 ± 0.22
2	7.797	251263 ± 0.04	3.92 ± 0.19
3	7.697	262256 ± 0.42	4.05 ± 0.12
Mean($\bar{x}$)	7.699	242256 ± 0.16	3.81 ± 0.31

Results are shown as the mean values of three replicates ± SD and are expressed in mg/g.

**Table 5:** Estimated percentage of betalains in the plant extract.

Peak #	Compounds	Percent contribution in TBC
1	Betanin	38.16 ± 0.31
2	Isobetanin	29.62 ± 0.22
3	Betanidin	20.12 ± 0.33
4	Isobetanidin	12.10 ± 1.2

**Table 6:** Betalains identified in *Beta vulgaris* L. peels and pomaces extract by LC-MS/MS.

Compound #	Compound name	*tR* [min]	Full-scan MS of [m/*z*]	MS/MS of [m/*z*]	Ions	Relative peak area (%) of betalains	Content estimates (mg/g) of dry weight
1.	2-*O*-Glucosyl-betanin	6.97	389.1	345.1	[*M* + *H*]^+^	4.02 ± 0.10	0.40 ± 0.10
2.	Betanin (betanidin 5-glucoside)	7.71	551.1	389.1	[*M* + *H*]^+^	43.12 ± 2.15	4.31 ± 2.15
3.	2-*O*-Glucosyl-isobetanin	7.98	389.1	345.1	[*M* + *H*]^+^	0.52 ± 0.08	0.05 ± 0.08
4.	Isobetanin (isobetanidin 5-glucoside)	8.81	551.1	389.1	[*M* + *H*]^+^	18.45 ± 2.20	1.85 ± 2.20
5	Betanidin	9.46	387.1	321.1, 267.2	[*M* + *H*]^+^	7.05 ± 0.03	0.71 ± 0.03
6.	Ferulic acid hexoside (4-glucoside)	9.68	355.1	315.1	[*M* − *H*]^−^	1.03 ± 0.02	0.10 ± 0.02
7.	17-Decarboxy-neobetanin	10.93	431.1	387.1, 267.2	[*M* + *H*]^+^	0.49 ± 0.07	0.05 ± 0.07
8.	Isobetanidin (17-decarboxy-betanidin)	11.67	345.1	267.1	[*M* + *H*]^+^	3.60 ± 1.26	0.36 ± 1.26
9.	Neobetanin	12.13	549.1	387.1	[*M* + *H*]^+^	3.24 ± 0.35	0.32 ± 0.35
10.	17-Decarboxy-betanin	13.72	507.2	343.1, 297.1	[*M* + *H*]^+^	0.35 ± 0.03	0.04 ± 0.03
11.	2,17-Bidecarboxy-betanin (isobetanin)	14.18	475.1	267.1	[*M* − *H*]^−^	1.59 ± 0.42	0.16 ± 0.42
12.	2,17-Bidecarboxy -neobetanin	14.78	461.2	299.1	[*M* + *H*]^+^	7.07 ± 0.02	0.71 ± 0.02
13.	Isovitexin (apigenin 6-C- glucoside)	14.86	433.2	343.1, 267.2	[*M* + *H*]^+^	2.54 ± 0.19	0.25 ± 0.19

Results are the mean values from five replicates ± S.D.

## Data Availability

The data used to support the findings of this study have been deposited in the respository found at https://ir.mu.ac.ke:8080/xmlui/handle/123456789/5622.

## Abbreviations

B. vulgaris L.: *Beta vulgaris* Linnaeus
RTD: Ready to drink
TBC: Total betalain content
TBPA: Total betalain peak area.
